# Urinary polycyclic aromatic hydrocarbon metabolites were associated with short sleep duration and self-reported trouble sleeping in US adults: data from NHANES 2005–2016 study population

**DOI:** 10.3389/fpubh.2023.1190948

**Published:** 2023-06-22

**Authors:** Lu Han, Qi Wang

**Affiliations:** Department of Obstetrics and Gynecology, First Affiliated Hospital, Xi'an Jiaotong University, Xi'an, China

**Keywords:** polycyclic aromatic hydrocarbon, short sleep duration, self-reported trouble sleeping, cross-sectional study, National Health and Nutrition Examination Survey

## Abstract

**Background:**

The aim of the current study was to investigate the link between human exposure to PAHs with short sleep duration (SSD) and self-reported trouble sleeping.

**Methods:**

A total of 9,754 participants and 9,777 participants obtained from NHANES 2005–2016 were included in this cross-sectional study about SSD and self-reported trouble sleeping, respectively. The association between urinary PAHs metabolites with the prevalence of SSD and self-reported trouble sleeping by the weighted multivariate logistic regression model, restricted cubic spline (RCS) curves, and weighted quantile sum (WQS) regression.

**Results:**

After adjusting for all covariates, 1-hydroxynapthalene, 2-hydroxynapthalene, 3-hydroxyfluorene, 2-hydroxyfluorene, 1-hydroxyphenanthrene, and 1-hydroxyphenanthrene demonstrated positive associations with SSD prevalence. Besides, 1-hydroxynapthalene, 2-hydroxynapthalene, 3-hydroxyfluorene, 2-hydroxyfluorene, 1-hydroxyphenanthrene, and 1-hydroxyphenanthrene exhibited positive associations with the prevalence of self-reported trouble sleeping following the adjustment for all covariates. RCS curves confirmed the non-linear associations between 1-hydroxynapthalene, 2-hydroxynapthalene, 3-hydroxyfluorene, 2-hydroxyfluorene, and 1-hydroxyphenanthrene with the prevalence of SSD, and 1-hydroxynapthalene, 3-hydroxyfluorene, and 2-hydroxyfluorene with the prevalence of self-reported trouble sleeping. The WQS results showed that mixed exposure to PAH metabolites had a significant positive association with the prevalence of SSD (OR: 1.087, 95% CI: 1.026, 1.152, *p* = 0.004) and self-reported trouble sleeping (OR: 1.190, 95% CI: 1.108, 1.278, *p* < 0.001).

**Conclusion:**

Urinary concentrations of PAH metabolites exhibited a close association with the prevalence of SSD and self-reported trouble sleeping in US adults. More emphasis should be placed on the importance of environmental effects on sleep health.

## Introduction

Sleeping is an active process that restores vitality and minimizes fatigue. One-third of the life of an individual is spent sleeping. Healthy sleep is important for physical and mental development, work effectiveness, cognitive status, and even mortality (1). Over the past 40 years, the duration Americans spend sleeping has been reduced by 1.5 to 2 h. According to the National Sleep Foundation, American adults slept an average of 6.9 h on workdays and 7.6 h on weekends in 2015 (2), compared to an average of 8.5 h in 1960 (3). Short sleep duration (SSD) and poor sleep quality have been linked to numerous adverse health outcomes, including obesity (4, 5), diabetes mellitus (6, 7), dementia (8, 9), asthma (10, 11), cardiovascular disorders (12, 13), metabolic syndrome (14, 15), among others. Several factors may impact the sleep, including age, psychological and physiological conditions, culture and environmental factors (16). Environmental pollution emerges as a new research orientation on factors affecting sleep status. For example, antimony (17), arsenic (18), pesticides (19), fluoride (20), and phthalates (21) all have been demonstrated to plays a negative role in sleep health.

Polycyclic aromatic hydrocarbons (PAHs), produced by the incomplete combustion of organic materials like tobacco, waste, fossil fuels, wood, and others, are the major constituents of air pollution (22). The general population might be subjected to PAHs in various potential ways such as inhalation (e.g., contaminated air from vehicle emissions, farm explosions, coke plants, power plants, and steel plants), ingestion (e.g., grilling, roasting, frying, or smoking foods, and contaminated water or milk), and skin contact (e.g., dust and soil) (23). With a half-life duration of shorter than 30 h, PAHs are processed by the liver in the human body and eliminated through urine and feces (24). Owing to the short half-life of PAHs, several metabolites in urine samples have been found to be effective biomarkers of PAH exposure (25). Accumulating evidence suggests that being exposed to PAH may have adverse impacts such as immunotoxicity (26, 27), carcinogenicity (28, 29), genotoxicity (30, 31), and teratogenicity (27).

Moreover, several recent reports have highlighted that exposure to PAH has a significant link to changes in brain structure (32–34), neurodegeneration, and neurodevelopmental inhibition (35, 36). Besides, exposure to PAH was confirmed to be associated with various brain diseases, including adverse cognitive function (37, 38), attention-deficit/hyperactivity disorder symptoms (39), conduct disorder (40), children’s intelligence quotient (41–43), and depression (44–46). Although numerous researchers have demonstrated that various PAH metabolites influence health status, there has not been much focus on the association of PAH exposure with SSD and self-reported trouble sleeping. Therefore, our research is a cross-sectional study that aimed at investigating the link between human exposure to PAHs with SSD and self-reported trouble sleeping based on 2005–2016 National Health and Nutrition Examination Survey (NHANES) data.

## Methods

### Study subjects

The NHANES program was performed to evaluate the nutritional and health status of American civilians using a complex, multistage sampling methodology. A substantial amount of data (including demographics, socioeconomic status, questions on diet and health, and medical history) were gathered via household interviews and biochemical evaluations of blood and urine samples at specific examination centers. The Centers for Disease Control and Prevention (CDC) provided details on the procedures, research design, and NHANES enrollment. Moreover, the National Center for Health Statistics Research Board reviewed and approved the NHANES protocol, followed by ensuring the consent of all the participants.

**Figure 1:**
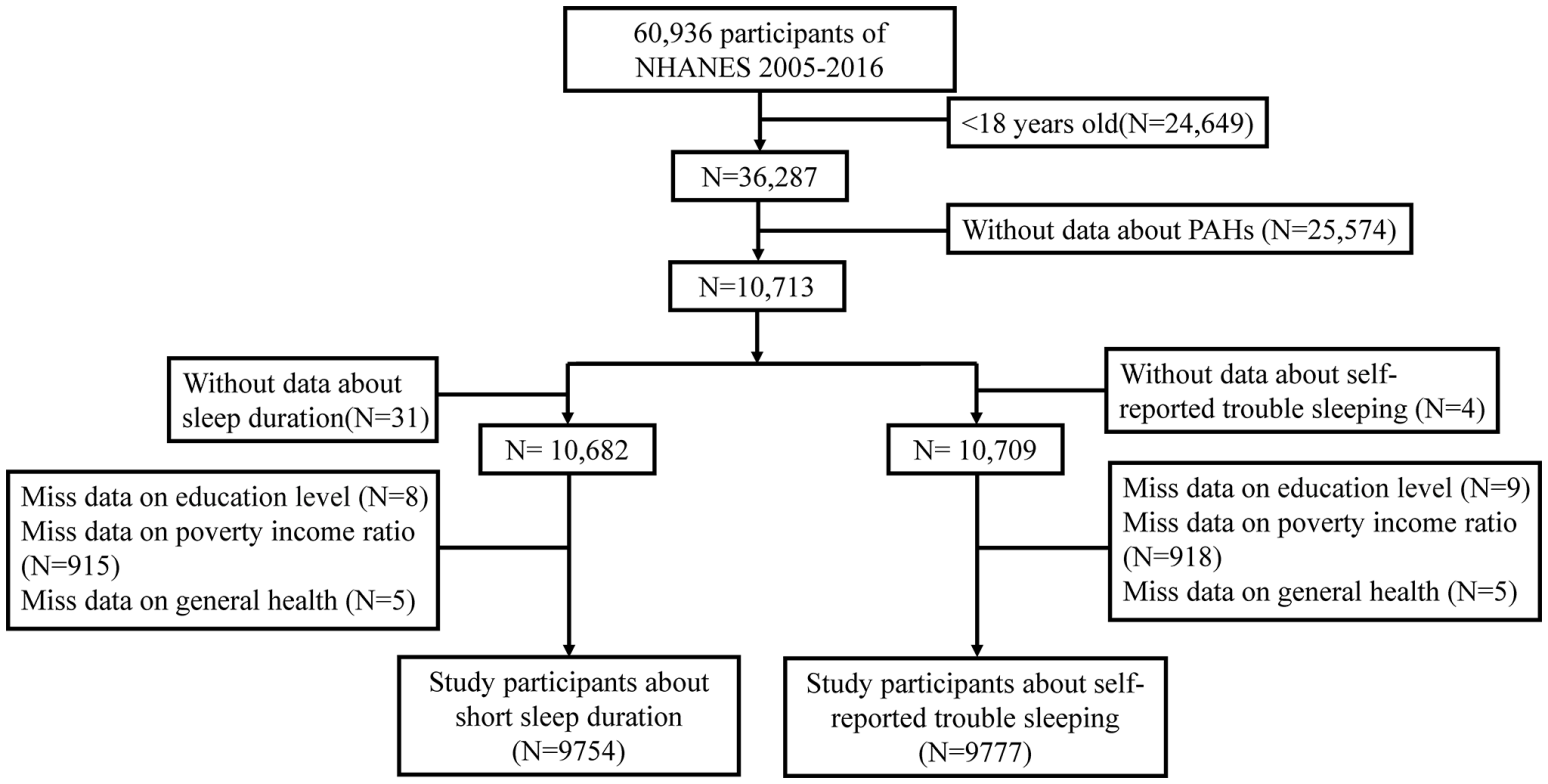
Flow chart of the participants’ selection.

Six NHANES waves, completed in succession between 2005 and 2016, were used to select the study sample. A total of 26,649 participants under 18 years of age and 25,574 individuals without PAH data were initially excluded from this study. The sample included 9,754 participants who met the eligibility criteria for the study on SSD after excluding 31 subjects without sleep duration data, 8 participants without education level data, 915 subjects lacking poverty income ratio (PIR) data, and 5 individuals without general health data. Additionally, 9,777 participants met the eligibility criteria for the study on self-reported trouble sleeping after excluding 4 subjects without sleep duration data, 9 participants without data on education level, 918 subjects lacking PIR data, and 5 individuals without general health data (Figure 1).

### Measurements of urinary PAH metabolites

The NHANES measured urinary mono-hydroxylated PAH metabolites across six NHANES cycles in a one-third subsample of participants aged 6 and above. The urinary mono-hydroxylated metabolites of PAHs were identified as stable biomarkers for multi-pathway exposure to PAHs. Several analytical techniques across different cycles were used to detect urine PAH metabolites. Specifically, capillary gas chromatography in combination with high-resolution mass spectrometry (GC–HRMS) was used in the 2005–2008 cycle, while isotope dilution capillary gas chromatography-tandem mass spectrometry (GC–MS/MS) was used in the 2009–2012 cycle. In the 2013–2016 cycle, isotope dilution high-performance liquid chromatography-MS/MS (online SPE-HPLC-MS/MS) was used in the detection process. Notably, there were variations in the urine PAH metabolites measured during the various cycles. However, six PAH metabolites, namely 1-hydroxynaphthalene, 2-hydroxynaphthalene, 3-hydroxyfluorene, 2-hydroxyfluorene, 1-hydroxyphenanthrene, and 1-hydroxypyrene were measured across all cycles and included in this study.

### Definition of short sleep duration and self-reported trouble sleeping

This study employed questions from the NHANES to record the duration of sleep of participants. Specifically, from 2005 to 2014, participants were asked about the duration that they sleep (in hours), while from 2015 to 2016, they were asked about how much they usually sleep at night on weekdays or workdays. Moreover, the expert consensus of the American Academy of Sleep Medicine and the Sleep Research Society defined SSD as a sleep duration of fewer than 7 h in an average 24-h period (47, 48). In addition, self-reported trouble sleeping was assessed by asking the participants “Have you ever told a doctor or other health professional that you have trouble sleeping?” Both yes and no were accepted as possible answers to this query.

### Covariates

A directed acyclic graph (DAG) drawn in DAGitty3.0 was used for the identification the minimum adjustment required for the confounder control (Supplementary Figure S1). This study considered several potential covariates, including age (years), gender (male or female), race/ethnicity (non-Hispanic White, non-Hispanic Black, Mexican Americans, other Hispanics, or other races), education (below high school, high school, or above high school), PIR (≤1.3, 1.3–3.5, or ≥ 3.5), and general health status. The PIR was derived by dividing household income by the poverty threshold and was categorized as low income (PIR <1.3), middle income (1.3 ≤ PIR <3.5), and high income (PIR ≥3.5) (49). General health status was assessed through the question “Would you say your health, in general, is …” and was divided into two categories: excellent, very good, or good versus fair or poor, as used in earlier studies (50, 51).

### Statistical analysis

Mean and standard deviation were used to report continuous variables with a normal distribution, whereas median and interquartile range were used to report skewed data. Categorical variables were reported as frequency and proportion. For categorical variables, the Chi-square test was utilized, while the *t*-test or Mann–Whitney *U* test was utilized for continuous variables, based on whether the data were normally distributed or not, to compare group differences between participants with SSD or self-reported trouble sleeping and the control group.

The odds ratios (ORs) and 95% confidence intervals (CIs) for PAHs associated with SSD or self-reported trouble sleeping were evaluated by employing the weighted multivariate logistic regression model. The statistical model comprised creatinine-adjusted PAHs as both a continuous variable (with log-transformation for non-normal distribution) and a categorical variable having the lowermost quartile as the reference group. The crude model was not adjusted, while Model I was adjusted for age and gender, along with adjusting Model II for age, gender, ethnic background, educational status, family PIR, and general health.

Furthermore, the current study utilized restricted cubic spline (RCS) curves with four knots to depict the dose–response relationship between PAHs and the prevalence of SSD and self-reported trouble sleeping following the adjustment for model variables (52). Additionally, the combined associations of all six PAH metabolites with SSD or self-reported trouble sleeping were assessed, and the relative contribution of each component in the mixture was determined for the positive association using weighted quantile sum (WQS) regression, a novel statistical method in environmental epidemiology (53). The WQS regression model was created to assess how mixed exposure affected health outcomes. All PAH metabolite concentrations were initially ranked in quartiles, and all data were then randomly divided into training and validation sets. The weighted index of each PAH metabolite represented its contribution to the positive association and was constrained to a range of 0–1, summing up to 1. A total of 1,000 bootstrap replicates were performed to estimate the effect size and 95% CIs based on previously published studies, followed by the random distribution of data into a 60% validation set and a 40% test set.

Sensitivity analysis was further performed to evaluate the robustness of our results. Adults with chronic conditions including (hypertension, cardiovascular disease, diabetes mellitus, and chronic obstructive pulmonary disease) were excluded to explore the associations. Participants were diagnosed as hypertension by: average systolic pressure ≥ 140 mmHg or average diastolic pressure ≥ 90 mmHg; or self-reported hypertension; or taking anti-hypertension drugs. Participants were diagnosed as diabetes mellitus by: doctor told you have diabetes; or glycohemoglobin HbA1() > 6.5%; or fasting glucose ≥7.0 mmoL/L; or random blood glucose ≥11.1 mmoL/L; or two-hour OGTT blood glucose ≥11.1 mmoL/L; or use of diabetes drugs or insulins. Participants were diagnosed as cardiovascular disease by doctor r told congestive heart failure, coronary heart disease, or angina pectoris, or heart attack, or stroke. Participants were diagnosed as COPD by: FEV1/FVC < 0.7; or ever told have emphysema; or use drugs including selective phosphodiesterase-4 inhibitors, mast cell stabilizers, leukotriene modifiers, inhaled corticosteroids. Besides, we conducted a primary study using train datasets (2005–2010 NHANES waves) for discovery of associations between urinary PAH metabolites with the prevalence of SSD and self-reported trouble sleeping, and used test datasets (2011–2016 NHANES waves) to replicate analysis.

The R software (version 4.1.3) was utilized for conducting all statistical analyses in the current study. The significance level for all statistical tests was set at two-tailed *p* < 0.05.

## Results

### General characteristics

This study focused on the link between urinary PAH metabolites and SSD. A total of 9,754 participants (represented 199.1 million non-institutionalized residents of United States) over 18 years of age were chosen for the current research, with 3,581 cases reporting SSD and 6,173 cases without SDD. The mean age was 45.71 ± 0.30 years, and females represented 50.34% of the sample. The majority of the participants were non-Hispanic white (42.86%), had at least high school education (51.15%), middle income (36.65%), and exhibited good, very good, or excellent general health status (77.64%). Participants with and without SSD differed significantly in terms of age, gender, ethnicity, education level, family PIR, and general health. Participants with SSD exhibited significantly higher concentrations of 1-hydroxynapthalene, 1-hydroxyphenanthrene, 1-hydroxypyrene, 2-hydroxynapthalene, 3-hydroxyfluorene, and 2-hydroxyfluorene compared to those without SSD (Table 1).

**Table 1:** Weighted characteristics of the study participants with and without short sleep duration.

Variable	Overall (*n* = 9,754)	No-SSD (*n* = 6,173)	SSD (*n* = 3,581)	*p*-value
Age [years, mean (SD)]	45.71(0.30)	45.99 (0.34)	45.19 (0.37)	0.03
*Gender (%)*				<0.0001
Female	4,910 (50.34)	3,175 (53.13)	1735 (48.07)	
Male	4,844 (49.66)	2,998 (46.87)	1846 (51.93)	
*Race/ethnicity (%)*				<0.0001
Mexican American	1,536 (15.75)	1,031 (8.47)	505 (8.01)	
Non-Hispanic Black	2,118 (21.71)	1,073 (8.69)	1,045 (16.70)	
Non-Hispanic White	4,181 (42.86)	2,841(70.82)	1,340 (62.13)	
Other Hispanic	905 (9.28)	570 (4.99)	335 (5.72)	
Other Racer	1,014 (10.4)	658 (7.03)	356 (7.44)	
*Educational status (%)*				0.01
Below high school	2,446 (25.08)	1,557 (16.82)	889 (17.11)	
High school	2,319 (23.77)	1,432 (21.93)	887 (25.08)	
Above high school	4,989 (51.15)	3,184 (61.25)	1805 (57.81)	
*PIR (%)*				< 0.001
≤1.3	3,224 (33.05)	1964 (20.74)	1,260 (24.80)	
1.3–3.5	3,575 (36.65)	2,252 (35.08)	1,323 (35.99)	
≥3.5	2,955 (30.3)	1957 (44.18)	998 (39.20)	
*General health (%)*				< 0.0001
Good/Very good/Excellent	7,573 (77.64)	4,921 (85.26)	2,652 (80.51)	
Fair/Poor	2,181 (22.36)	1,252 (14.74)	929 (19.49)	
*Country of birth (%)*				0.63
Born in 50 US states or Washington, DC	7,187 (73.7)	4,467 (83.35)	2,720 (83.77)	
Others	2,565 (26.3)	1705 (16.65)	860 (16.23)	
*Citizenship status (%)*				0.01
Citizen by birth or naturalization	8,414 (86.37)	5,225 (90.74)	3,189 (92.44)	
Not a citizen of the US	1,328 (13.63)	939 (9.26)	389 (7.56)	
*Exposures [μg/L, median (IQR)]*				
1-Hydroxynapthalene	1664.90 (688.00, 5976.00)	1559.00 (650.00, 5112.00)	1907.00 (771.00, 7506.00)	< 0.0001
2-Hydroxynapthalene	4128.30 (1802.00, 9740.00)	3911.00 (1639.00, 9246.00)	4845.00 (2092.00, 10882.50)	< 0.0001
3-Hydroxyfluorene	81.20 (38.00, 247.00)	75.00 (35.90, 208.00)	94.00 (43.00, 335.00)	< 0.0001
2-Hydroxyfluorene	221.20 (104.00, 559.00)	203.80 (97.00, 480.00)	260.00 (120.70, 694.00)	< 0.0001
1-Hydroxyphenanthrene	127.00 (68.00, 232.00)	119.60 (64.00, 222.00)	139.00 (74.00, 250.00)	< 0.0001
1-Hydroxypyrene	108.60 (49.50, 218.50)	103.00 (49.50, 206.10)	118.00 (54.80, 240.00)	< 0.0001

SSD, short sleep duration; PIR, family poverty income ratio; IQR, interquartile range.

Additionally, the study focused on the association of urinary PAH metabolites with self-reported trouble sleeping. This study comprised 9,777 participants (represented 199.5 million non-institutionalized residents of United States), with 2,322 cases of self-reported trouble sleeping and 7,455 cases with no self-reported trouble sleeping. The mean age was 54.72 ± 0.30 years, and females accounted for 50.36%. Most of the subjects were non-Hispanic white (42.88%), had an education above high school (51.11%), middle income (36.61%), and presented good, very good, or excellent general health status (77.53%). Individuals with and without self-reported trouble sleeping differed considerably in terms of age, gender, ethnicity, educational attainment, family PIR, and general health. Participants with self-reported trouble sleeping exhibited significantly higher concentrations of 1-hydroxynapthalene, 2-hydroxynapthalene, 3-hydroxyfluorene, 2-hydroxyfluorene, 1-hydroxyphenanthrene, and 1-hydroxypyrene compared to those without self-reported trouble sleeping (Table 2). Moreover, the percentiles of the urinary PAHs in both creatinine adjusted and unadjusted in study about SSD and self-reported trouble sleeping were represented in Supplementary Tables S1, S2.

**Table 2:** Weighted characteristics of the study participants with and without self-reported trouble sleeping.

Variable	Overall (*n* = 9,777)	No-trouble sleeping (*n* = 7,455)	Trouble sleeping (*n* = 2,322)	*p*-value
*Age [years, mean (SD)]*	45.72 (0.30)	44.50 (0.34)	49.22 (0.46)	< 0.0001
*Gender (%)*				<0.0001
Female	4,924 (50.36)	3,605 (49.28)	1,319 (57.51)	
Male	4,853 (49.64)	3,850 (50.72)	1,003 (42.49)	
*Race/ethnicity (%)*				<0.0001
Mexican American	1,540 (15.75)	1,271 (9.42)	269 (5.19)	
Non-Hispanic Black	2,121 (21.69)	1,665 (11.94)	456 (10.04)	
Non-Hispanic White	4,192 (42.88)	2,966 (64.85)	1,226 (76.28)	
Other Hispanic	907 (9.28)	719 (5.86)	188 (3.46)	
Other Racer	1,017 (10.4)	834 (7.94)	183 (5.03)	
*Educational status (%)*				0.02
Below high school	2,458 (25.14)	1933 (17.83)	525 (14.50)	
High school	4,997 (51.11)	3,768 (59.53)	1,229 (61.50)	
Above high school	2,322 (23.75)	1754 (22.64)	568 (24.00)	
*PIR (%)*				0.02
≤1.3	3,240 (33.14)	2,424 (22.02)	816 (22.79)	
1.3–3.5	3,579 (36.61)	2,793 (36.28)	786 (32.78)	
≥3.5	2,958 (30.25)	2,238 (41.70)	720 (44.44)	
*General Health (%)*				< 0.0001
Good/Very good/Excellent	7,580 (77.53)	6,049 (86.55)	1,531 (74.83)	
Fair/Poor	2,197 (22.47)	1,406 (13.45)	791 (25.17)	
*Country of birth (%)*				< 0.0001
Born in 50 US states or Washington, DC	7,205 (73.71)	5,281 (81.14)	1924 (90.20)	
Others	2,570 (26.29)	2,172 (18.86)	398 (9.80)	
*Citizenship status (%)*				< 0.0001
Citizen by birth or naturalization	8,435 (86.38)	6,257 (89.58)	2,178 (96.36)	
Not a citizen of the US	1,330 (13.62)	1,187 (10.42)	143 (3.64)	
*Exposures [μg/L, median (IQR)]*				
1-Hydroxynapthalene	1666.50 (690.00, 5986.00)	1600.00 (665.20, 5311.20)	1976.90 (764.00, 7760.70)	< 0.0001
2-Hydroxynapthalene	4135.0 0 (1805.00, 9750.00)	3996.00 (1730.00, 9463.00)	4734.00 (1970.00, 10657.00)	0.005
3-Hydroxyfluorene	81.20 (38.00, 247.70)	80.30 (38.00, 223.00)	83.50 (38.40, 340.00)	0.02
2-Hydroxyfluorene	221.20 (104.10, 561.00)	218.50 (104.00, 509.60)	230.50 (106.00, 675.80)	0.01
1-Hydroxyphenanthrene	127.00 (68.00, 233.00)	126.00 (67.00, 228.00)	129.00 (69.00, 245.00)	0.08
1-Hydroxypyrene	108.80 (49.50, 218.50)	108.00 (49.50, 213.00)	110.00 (49.50, 233.30)	0.38

### Measurements of PAH metabolites and their correlations

**Figure 2:**
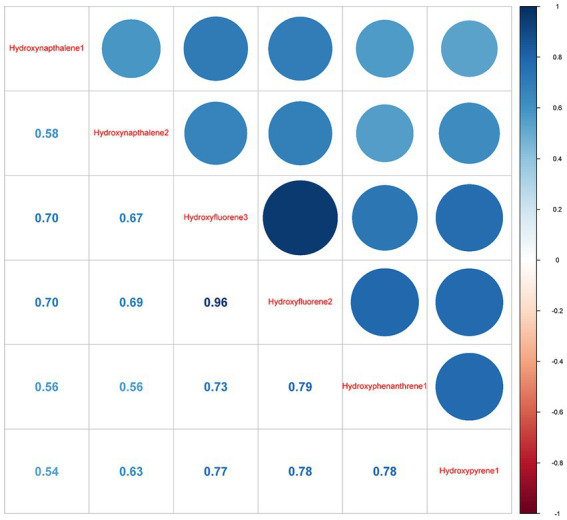
Pearson correlations between log-transformed concentrations of six urinary creatinine-corrected PAH metabolites. Hydroxynapthalene1, 1-Hydroxynapthalene; Hydroxynapthalene2, 2-Hydroxynapthalene; Hydroxyfluorene3, 3-Hydroxyfluorene, Hydroxyfluorene2, 2-Hydroxyfluorene; Hydroxyphenanthrene1, 1-Hydroxyphenanthrene; Hydroxypyrene1, 1-Hydroxypyrene.

Pearson correlation findings revealed a close association of 3-hydroxyfluorene with 2-hydroxyfluorene (r = 0.96), followed by 2-hydroxyfluorene and 1-hydroxyphenanthrene with a correlation coefficient of 0.79 (Figure 2).

### Association between single PAH metabolites with sleep health

**Figure 3:**
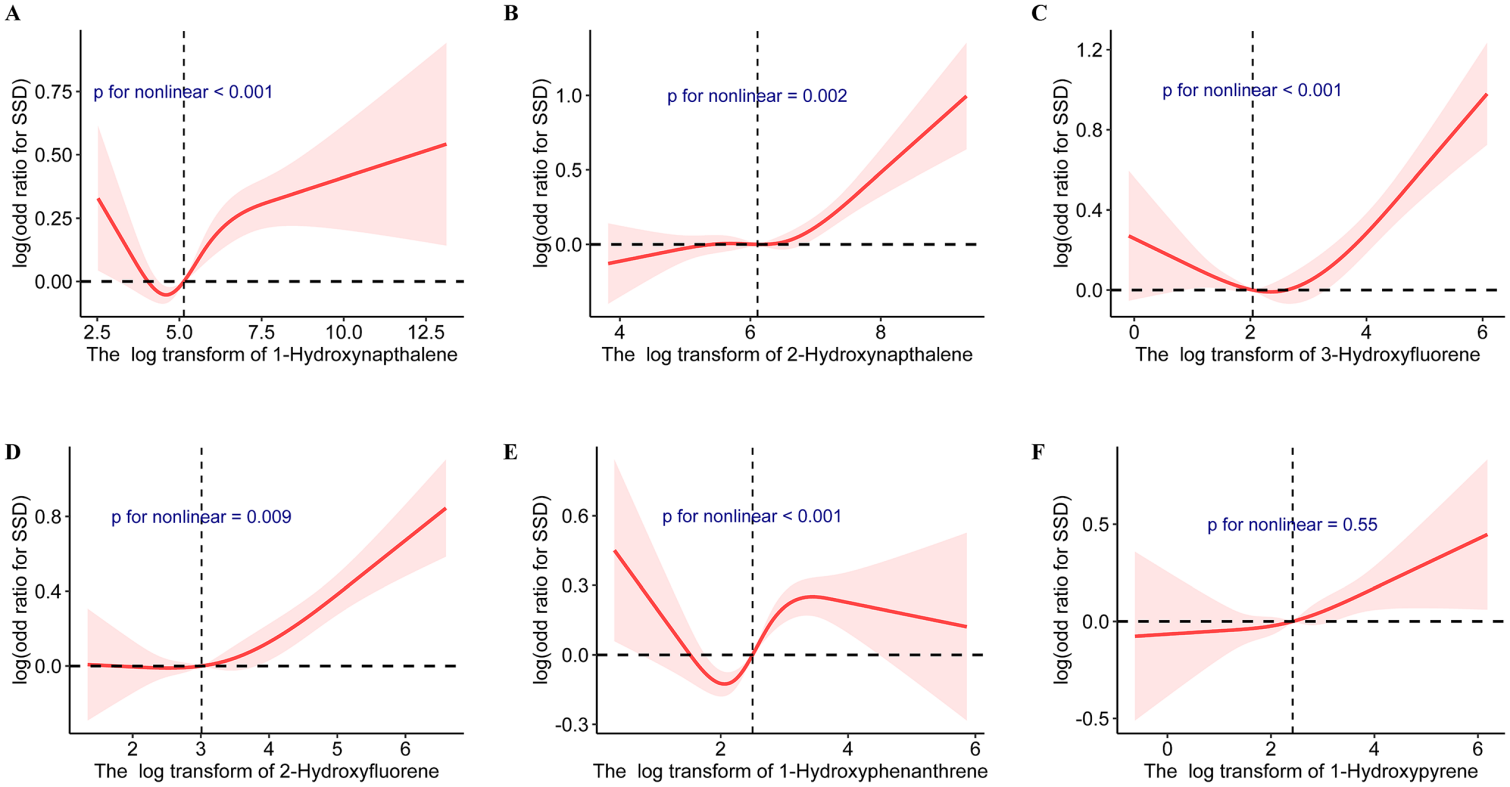
Cubic splines for the associations of urinary PAH metabolites with the prevalence of short sleep duration. Model adjusted for age, gender, race/ethnicity, educational status, family poverty income ratio, and general health. **(A)** 1-Hydroxynapthalene; **(B)** 2-Hydroxynapthalene; **(C)** 3-Hydroxyfluorene; **(D)** 2-Hydroxyfluorene; **(E)** 1-Hydroxyphenanthrene; **(F)** 1-Hydroxypyrene.

Table 2 presents the outcomes of weighted generalized logistic regression models adjusting for various covariates to evaluate the association of single PAH metabolites (continuous) with SSD prevalence. For the crude model, the current investigation observed substantial correlations among all PAH metabolites except 1-hydroxyphenanthrene and SSD. Following adjustment for age and gender, 1-hydroxyphenanthrene was also significantly linked to the prevalence of SSD (*p* = 0.02). After further adjusting for race/ethnicity, educational status, family PIR, and general health, 1-hydroxynapthalene (OR: 1.08; 95% CI: 1.04, 1.11, *p* < 0.0001), 2-hydroxynapthalene (OR: 1.14; 95% CI: 1.06, 1.21, *p* < 0.001), 3-hydroxyfluorene (OR: 1.11; 95% CI: 1.06, 1.17, *p* < 0.0001), 2-hydroxyfluorene (OR: 1.16; 95% CI: 1.10, 1.23, *p* < 0.0001), 1-hydroxyphenanthrene (OR: 1.14; 95% CI: 1.06, 1.24, *p* < 0.001), and 1-hydroxyphenanthrene (OR: 1.08; 95% CI: 1.01, 1.16, *p* < 0.001) demonstrated positive associations with SSD prevalence (Table 3). When the corresponding PAH metabolites were further divided into quantile groups, and the lowest quantile was set as a reference, significant positive associations were observed between 1-hydroxynapthalene (OR: 1.33; 95% CI: 1.16, 1.52, *p* for trend <0.001), 2-hydroxynapthalene (OR: 1.38; 95% CI: 1.14, 1.65, *p* for trend = 0.002), 3-hydroxyfluorene (OR: 1.24; 95% CI: 1.05, 1.47, *p* for trend = 0.028), 2-hydroxyfluorene (OR: 1.41; 95% CI: 1.21, 1.16, *p* for trend <0.001), 1-hydroxyphenanthrene (OR: 1.37; 95% CI: 1.17, 1.60, *p* for trend <0.0001), and 1-hydroxyphenanthrene (OR: 1.24; 95% CI: 1.06, 1.47, *p* for trend = 0.011) in the highest quartile with the prevalence of SSD (Table 4). RCS curves were used to model the connection between log-transformed concentrations of PAH metabolites and SSD and to detect any potential nonlinearity. The results confirmed the non-linear associations between 1-hydroxynapthalene (*p* for nonlinear <0.001), 2-hydroxynapthalene (*p* for nonlinear = 0.002), 3-hydroxyfluorene (*p* for nonlinear <0.001), 2-hydroxyfluorene (*p* for nonlinear = 0.009), and 1-hydroxyphenanthrene (*p* for nonlinear <0.001) with the prevalence of SSD (Figure 3).

**Table 3:** Comparison between different models of the weighted relationship between log-transformed urinary PAH metabolites and prevalence of short sleep duration.

Chemicals (μg/mg creatinine)	Crude model OR (95%CI)	*p*-value	Model I OR (95%CI)	*p*-value	Model II OR (95%CI)	*p*-value
1-Hydroxynapthalene	1.07 (1.04, 1.11)	**<0.0001**	1.08 (1.05, 1.12)	**<0.0001**	1.08 (1.04, 1.11)	**<0.0001**
2-Hydroxynapthalene	1.15 (1.08, 1.21)	**<0.0001**	1.16 (1.10, 1.23)	**<0.0001**	1.14 (1.06, 1.21)	**<0.001**
3-Hydroxyfluorene	1.14 (1.08, 1.19)	**<0.0001**	1.13 (1.08, 1.19)	**<0.0001**	1.11 (1.06, 1.17)	**<0.0001**
2-Hydroxyfluorene	1.18 (1.11, 1.24)	**<0.0001**	1.18 (1.11, 1.24)	**<0.0001**	1.16 (1.10, 1.23)	**<0.0001**
1-Hydroxyphenanthrene	1.07 (0.99, 1.15)	0.08	1.09 (1.01, 1.18)	**0.02**	1.14 (1.06, 1.24)	**<0.001**
1-Hydroxypyrene	1.07 (1.00, 1.14)	**0.03**	1.08 (1.01, 1.15)	**0.02**	1.08 (1.01, 1.16)	**0.02**

Crude model: no adjustment; Model I: only adjusted for age and gender; Model II: adjusted for age, gender, race/ethnicity, educational status, family poverty income ratio, and general health; OR, odds ratio; CI, confidence interval. The bold indicates *p* - value less than 0.05.

**Table 4:** Comparison between different models of the weighted relationship between log-transformed urinary PAH metabolites and prevalence of self-reported trouble sleeping.

Chemicals (μg/mg creatinine)	Crude model OR (95%CI)	*p*-value	Model I OR (95%CI)	*p*-value	Model II OR (95%CI)	*p*-value
1-Hydroxynapthalene	1.17 (1.13, 1.21)	**<0.0001**	1.15 (1.11, 1.18)	**<0.0001**	1.12 (1.08, 1.16)	**<0.0001**
2-Hydroxynapthalene	1.19 (1.11, 1.27)	**<0.0001**	1.20 (1.13, 1.28)	**<0.0001**	1.20 (1.12, 1.28)	**<0.001**
3-Hydroxyfluorene	1.12 (1.08, 1.17)	**<0.0001**	1.15 (1.11, 1.20)	**<0.0001**	1.12 (1.07, 1.17)	**<0.0001**
2-Hydroxyfluorene	1.17 (1.11, 1.22)	**<0.0001**	1.19 (1.13, 1.25)	**<0.0001**	1.14 (1.08, 1.21)	**<0.0001**
1-Hydroxyphenanthrene	1.21 (1.13, 1.30)	**<0.0001**	1.16 (1.08, 1.24)	**<0.0001**	1.09 (1.02, 1.18)	**0.02**
1-Hydroxypyrene	1.12 (1.05, 1.20)	**0.001**	1.16 (1.08, 1.25)	**<0.0001**	1.13 (1.04, 1.22)	**0.003**

Crude model: no adjustment; Model I: only adjusted for age and gender; Model II: adjusted for age, gender, race/ethnicity, educational status, family poverty income ratio, and general health; OR: odds ratio; CI, confidence interval. The bold indicates *p* - value less than 0.05.

Table 5 depicts the findings of weighted generalized logistic regression models adjusting for various covariates to evaluate the association of a single PAH metabolite (continuous) with the prevalence of self-reported trouble sleeping. Following the adjustment for all covariates, 1-hydroxynapthalene (OR: 1.12; 95% CI: 1.08, 1.16, *p* < 0.0001), 2-hydroxynapthalene (OR: 1.20; 95% CI: 1.12, 1.28, *p* < 0.001), 3-hydroxyfluorene (OR: 1.12; 95% CI: 1.07, 1.17, *p* < 0.0001), 2-hydroxyfluorene (OR: 1.14; 95% CI: 1.08, 1.21, *p* < 0.0001), 1-hydroxyphenanthrene (OR: 1.09; 95% CI: 1.02, 1.18, *p* = 0.02), and 1-hydroxyphenanthrene (OR: 1.13; 95% CI: 1.04, 1.22, *p* = 0.003) exhibited positive associations with the prevalence of self-reported trouble sleeping (Table 5). After further classifying corresponding PAH metabolites into quantile groups and using the lowest quantile as a reference, significant positive associations were observed between 1-hydroxynapthalene (OR: 1.44; 95% CI: 1.19, 1.73, *p* for trend <0.0001), 2-hydroxynapthalene (OR: 1.50; 95% CI: 1.24, 1.81, *p* for trend <0.0001), 3-hydroxyfluorene (OR: 1.34; 95% CI: 1.12, 1.60, *p* for trend =0.001), 2-hydroxyfluorene (OR: 1.32; 95% CI: 1.13, 1.54, *p* for trend <0.001), and 1-hydroxyphenanthrene (OR: 1.36; 95% CI: 1.12, 1.65, *p* for trend =0.012) in the highest quartile with the prevalence of self-reported trouble sleeping (Table 6). RCS curves confirmed the non-linear associations between 1-hydroxynapthalene (*p* for nonlinear =0.012), 3-hydroxyfluorene (*p* for nonlinear =0.007), and 2-hydroxyfluorene (*p* for nonlinear =0.007) with the prevalence of self-reported trouble sleeping (Supplementary Figure S2).

**Table 5:** Association between quartiles of single PAH metabolite and prevalence of short sleep duration.

Chemicals (μg/mg creatinine)	Q1 OR (95%CI)	Q2 OR (95%CI)	Q3 OR (95%CI)	Q4 OR (95%CI)	*p* for trend
*1-Hydroxynapthalene*					**<0.001**
SDD/non-SSD	911/1530	841/1596	841/1597	988/1450	
Model	1.00 (reference)	0.92 (0.77, 1.10)	0.88 (0.75, 1.03)	**1.33 (1.16, 1.52)**	
*2-Hydroxynapthalene*					**0.002**
SDD/non-SSD	872/1566	878/1559	870/1571	961/1477	
Model	1.00 (reference)	1.07 (0.89, 1.28)	1.01 (0.86, 1.20)	**1.38 (1.14, 1.65)**	
*3-Hydroxyfluorene*					**0.028**
SDD/non-SSD	873/1564	841/1599	854/1584	1013/1426	
Model	1.00 (reference)	0.89 (0.75, 1.06)	0.87 (0.73, 1.04)	**1.24 (1.05, 1.47)**	
*2-Hydroxyfluorene*					**<0.001**
SDD/non-SSD	856/1582	841/1598	868/1570	1016/1423	
Model	1.00 (reference)	0.99 (0.86, 1.13)	1.00 (0.84, 1.19)	**1.41 (1.21, 1.66)**	
*1-Hydroxyphenanthrene*					**<0.0001**
SDD/non-SSD	928/1511	817/1616	875/1568	961/6163	
Model	1.00 (reference)	0.98 (0.85, 1.14)	1.08 (0.93, 1.24)	**1.37 (1.17, 1.60)**	
*1-Hydroxypyrene*					**0.011**
SDD/non-SSD	913/1525	877/1526	821/1619	970/1467	
Model	1.00 (reference)	0.99 (0.83, 1.18)	0.97 (0.82, 1.15)	**1.24 (1.06, 1.47)**	

Model adjusted for age, gender, race/ethnicity, educational status, family poverty income ratio, and general health.

Q1, Q2, Q3, and Q4 represented the first, the second, the third, and the fourth quartile of log-transformed urinary PAH metabolites.

SSD, short sleep duration; OR, odds ratio; CI, confidence interval. The bold indicates *p* - value less than 0.05.

**Table 6:** Association between quartiles of single PAH metabolite and prevalence of self-reported trouble sleeping.

Chemicals (μg/mg creatinine)	Q1 OR (95%CI)	Q2 OR (95%CI)	Q3 OR (95%CI)	Q4 OR (95%CI)	*p* for trend
*1-Hydroxynapthalene*					**<0.0001**
STS/non-STS	491/1954	521/1920	571/1879	739/1702	
Model	1.00 (reference)	1.03 (0.84, 1.26)	1.13 (0.91, 1.39)	**1.44 (1.19, 1.73)**	
*2-Hydroxynapthalene*					**<0.0001**
STS/non-STS	528/1915	536/1905	560/1889	698/1746	
Model	1.00 (reference)	1.09 (0.90, 1.32)	**1.24 (1.03, 1.50)**	**1.50 (1.24, 1.81)**	
*3-Hydroxyfluorene*					**0.001**
STS/non-STS	554/1892	510/1934	527/1915	731/1714	
Model	1.00 (reference)	0.90 (0.74, 1.10)	0.96 (0.79, 1.16)	**1.34 (1.12, 1.60)**	
*2-Hydroxyfluorene*					**<0.001**
STS/non-STS	524/1901	513/1933	529/1914	738/1707	
Model	1.00 (reference)	0.90 (0.74, 1.09)	0.95 (0.80, 1.13)	**1.32 (1.13, 1.54)**	
*1-Hydroxyphenanthrene*					**0.041**
STS/non-STS	488/1954	549/1896	593/1851	692/1754	
Model	1.00 (reference)	1.00 (0.82, 1.22)	1.05 (0.88, 1.26)	1.18 (0.98, 1.42)	
*1-Hydroxypyrene*					**0.012**
STS/non-STS	539/1904	555/1891	538/1905	690/1755	
Model	1.00 (reference)	1.16 (0.98, 1.39)	1.04 (0.86, 1.26)	**1.36 (1.12, 1.65)**	

Self-reported trouble sleeping, STS.

Model adjusted for age, gender, race/ethnicity, educational status, family poverty income ratio, and general health.

Q1, Q2, Q3, and Q4 represented the first, the second, the third, and the fourth quartile of log-transformed urinary PAH metabolites.

SSD, short sleep duration; OR, odds ratio; CI, confidence interval.

### Sensitivity analysis

After excluding 3,792 participants with hypertension, 179 participants without sufficient data to diagnose diabetes mellitus, 460 participants with diabetes mellitus, 532 participants without sufficient data to diagnose cardiovascular disease, 142 participants with cardiovascular disease, 107 participants with COPD, 4542 participants without chronic conditions including (hypertension, cardiovascular disease, diabetes mellitus, and chronic obstructive pulmonary disease) were enrolled in this sensitivity analysis to explore the association between association between urinary PAH metabolites with prevalence of SSD. All log-transformed PAH metabolites are positively correlated with SSD using weighted logistic regression model analysis with confounding variables (Supplementary Table S3). After excluding (3,804 participants with hypertension, 180 participants without sufficient data to diagnose diabetes mellitus, 462 participants with diabetes mellitus), 532 participants without sufficient data to diagnose cardiovascular disease, 143 participants with cardiovascular disease, 107 participants with COPD, 4549 participants without chronic conditions including (hypertension, cardiovascular disease, diabetes mellitus, and chronic obstructive pulmonary disease) were enrolled in this sensitivity analysis to explore the association between association between urinary PAH metabolites with prevalence of self-reported trouble sleeping. All log-transformed PAH metabolites are also positively correlated with self-reported trouble sleeping using weighted logistic regression model analysis with confounding variables (Supplementary Table S4). After cutting the enrolled participants as test and train datasets, the individual associations between prevalence of SSD with concentrations of urinary 1-Hydroxynapthalene, 2-Hydroxynapthalene, 3-Hydroxyfluorene, and 2-Hydroxyfluorene in 2005–2010 NHANES waves were also replicated in 2011–2016 NHANES waves (Supplementary Table S5). Besides, the individual associations between prevalence of self-reported trouble sleeping with concentrations of urinary 1-Hydroxynapthalene, 2-Hydroxynapthalene, 3-Hydroxyfluorene, and 2-Hydroxyfluorene in 2005–2010 NHANES waves were also replicated in 2011–2016 NHANES waves (Supplementary Table S6).

### Association between multiple PAH metabolites with sleep health

**Figure 4:**
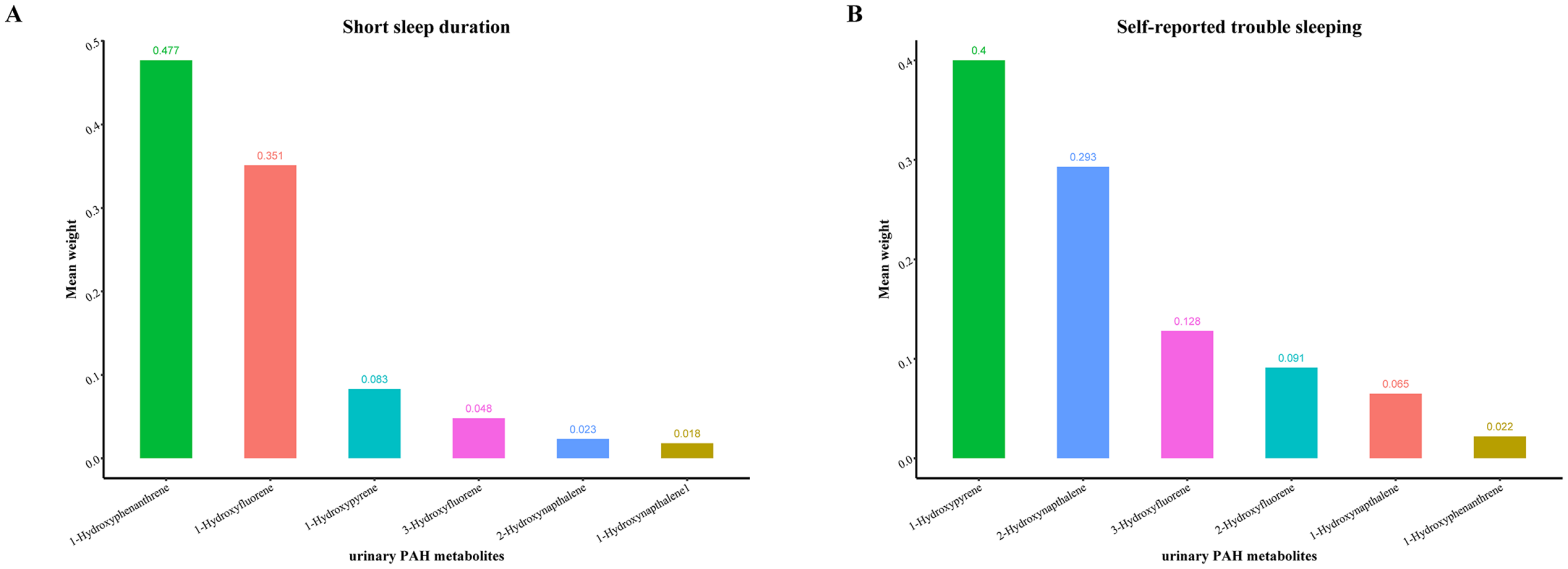
The weights of each urinary PAH metabolite in positive WQS model regression index for the prevalence of **(A)** short sleep duration; **(B)** self-reported trouble sleeping. Model adjusted for age, gender, race/ethnicity, educational status, family poverty income ratio, and general health. Hydroxynapthalene1, 1-Hydroxynapthalene; Hydroxynapthalene2, 2-Hydroxynapthalene; Hydroxyfluorene3, 3-Hydroxyfluorene, Hydroxyfluorene2, 2-Hydroxyfluorene; Hydroxyphenanthrene1, 1-Hydroxyphenanthrene; Hydroxypyrene1, 1-Hydroxypyrene.

The WQS was employed to derive the combined effect of metabolites on SSD prevalence and self-reported trouble sleeping (Figure 3). The results revealed that mixed exposure to PAH metabolites had a significant positive link to the SSD prevalence (OR: 1.019; 95% CI: 1.006, 1.032, *p* = 0.004), with the 3-hydroxyfluorene having the highest weight (weight = 0.627) (Figure 4A). In addition, WQS results revealed that increased mixed exposure of PAH metabolites was linked to the higher prevalence of self-reported trouble sleeping (OR: 1.031; 95% CI: 1.018, 1.044, *p* < 0.001), and the 1-hydroxypyrene had the highest weights (weight = 0.382) (Figure 4B).

## Discussion

The current research is thought to determine the link between urinary PAH levels and the prevalence of sleep health. The study observed that all single and multiple PAH metabolites were positively linked to the prevalence of SSD and self-reported trouble sleeping.

A previous study conducted using data from NHANES 2005–2006 discovered significant associations between higher urine levels of polyaromatic hydrocarbons, including 2-hydroxyfluorene, 9-hydroxyfluorene, 1-hydroxypyrene, 2-hydroxyphenanthrene and leg cramps during sleep. Additionally, urinary 2-hydroxyfluorene, 3-hydroxyfluorene, and 1-hydroxypyrene were observed to have a substantial link to leg jerks during sleep (54). On the contrary, the current study focused on sleep health concerning self-reported trouble sleeping and SSD. Moreover, it involved a relatively large sample size obtained from the NHANES database from 2005 to 2016. Besides, Zhang et al. explored the relationship between polycyclic aromatic hydrocarbons exposure and sleep quality in workers from a coking plant. A total of 632 employed workers in the coking plant were set as the exposed group, and 477 employed workers in the water-pump plant as the control group. They found the concentration of 12 polycyclic aromatic hydrocarbons in the peripheral blood of the exposure group was significantly higher than that of the control group. Besides, the detection rate of sleep disorder in the exposure group was higher than that in the control group (55). Unlike the above study, our study focused the linear and non-linear association between urinary PAHs with SSD and self-reported trouble sleeping in the general US. adults.

Numerous factors can impact sleep, including sleep environment, stress and anxiety, diet and exercise, and genetics (56). In addition to these factors, it has been reported that air pollution and exposure to certain chemicals can significantly affect sleep patterns and cause sleep disorders. For instance, higher annual NO2 and PM2.5 exposure levels have been linked to greater odds of sleep apnea (57). According to a population-based study carried out in the urban areas of Northern Taiwan, PM2.5 has been confirmed to be associated with sleep-disordered breathing (58). Notably, children who are exposed to traditional biomass fuel stoves display sleep apnea-related symptoms more frequently (59). Furthermore, exposure to specific chemicals has emerged as a topic of concern for sleep health. The central nervous system has been shown to be impacted by heavy metal exposure, including lead, mercury, antimony, and cadmium, which can also disturb the sleep–wake cycle. Higher levels of these metals in human bodies make individuals more susceptible to sleep disorders (17, 54, 60, 61). Additionally, exposure to certain pesticides has been associated with difficulty falling asleep, frequent night awakenings, and decreased overall sleep quality (19, 62–64). This study focused on the correlation between urinary PAH metabolites with self-reported sleep problems and SSD.

The underlying mechanisms behind the inverse relationship between urinary PAH metabolites and sleep health need to be investigated further. First, exposure to PAH is associated with higher levels of tumor necrosis factor-alpha and interleukin-1 beta (65–67). It has been observed that these two cytokines indirectly and directly impact the regulation of sleep architecture and duration by acting on neurons and stimulating the activity of molecules including gonadotropin-releasing hormone receptor, adenosine, and prostaglandin D2 (68, 69). Second, exposure to PAH is linked to elevated levels of nitric oxide (NO) and NO production (67, 70, 71). NO has been reported to play a role in the homeostatic regulation of REM sleep and, to a lesser extent, slow-wave sleep (72). Third, being exposed to PAHs has been linked to oxidative stress (73–75), which can disrupt sleep homeostasis through several mechanisms including the oxidative inactivation of cGMP which is responsible for mediating the influence of NO, inactivating the key proteins involved in sleep regulation, the modulation of ATP exocytosis from astrocytes, and NMDA-mediated neurotransmission (68, 76, 77). However, these mechanisms need to be confirmed further using *in vitro* and *in vivo* experiments.

Furthermore, the current research exhibited multiple strengths. First, a considerably large sample size was used in this investigation, contributing to the consistency of the results. Second, this was the first investigation to determine the link between PAH exposure with SSD and self-reported trouble sleeping. Last, several methods, including survey-weighted generalized logistic regressions, RCS curves, and WQS regression models, were employed for exploring the individual and overall impacts of PAH exposure on sleep health. This study also had some limitations. First, the data on urinary PAH metabolites were single measurements, which may not reflect the effects of participants’ long-term exposure. Second, self-reported trouble sleeping was not accurately measured by instruments or scales, which might have caused some errors. Third, although many potential confounders were enrolled, there remained residual confounders. Fourth, although the associations between urinary concentrations of PAH metabolites with the prevalence of SSD and self-reported trouble sleeping were detected in train datasets (2005–2010 NHANES waves) and also replicated in test datasets (2011–2016 NHANES waves), there still existed some bias caused by multiple NHANES survey rounds. Last, it was difficult to determine the directionality of associations for the nature of cross-sectional studies. However, the study did not intend to investigate the influence of sleep health on urinary PAH metabolites.

## Conclusion

Urinary concentrations of PAH metabolites exhibited a close association with the prevalence of SSD and self-reported trouble sleeping in US adults. More emphasis should be placed on the importance of environmental effects on sleep health.

## Data availability statement

The original contributions presented in the study are included in the article/Supplementary material, further inquiries can be directed to the corresponding author.

## Author contributions

LH: software and writing–original draft preparation. QW: conceptualization, methodology, and writing–reviewing and editing. All authors contributed to the article and approved the submitted version.

## Conflict of interest

The authors declare that the research was conducted in the absence of any commercial or financial relationships that could be construed as a potential conflict of interest.

## Publisher’s note

All claims expressed in this article are solely those of the authors and do not necessarily represent those of their affiliated organizations, or those of the publisher, the editors and the reviewers. Any product that may be evaluated in this article, or claim that may be made by its manufacturer, is not guaranteed or endorsed by the publisher.
