# Supplementary material for: Urinary polycyclic aromatic hydrocarbon metabolites were associated with short sleep duration and self-reported trouble sleeping in US adults: data from NHANES 2005–2016 study population
Source: Front Public Health. 2023 Jun 22;11:1190948. doi: 10.3389/fpubh.2023.1190948 (PMC10325832; doi:10.3389/fpubh.2023.1190948)

Table S1. The percentiles of the urinary PAHs in both creatinine adjusted and unadjusted in study about short sleep duration.

Variable	25th percentile	50th percentile	75th percentile
**Unadjusted urinary PAHs (μg/L)**			
1-Hydroxynapthalene	735.00	1769.00	6345.83
2-Hydroxynapthalene	2041.25	4700.00	10680.00
3-Hydroxyfluorene	40.00	84.10	245.00
2-Hydroxyfluorene	111.00	231.00	577.00
1-Hydroxyphenanthrene	67.00	125.00	232.00
1-Hydroxypyrene	49.50	110.00	227.00
**Creatinine adjusted urinary PAHs (μg/mg creatinine)**			
1-Hydroxynapthalene	77.83	169.81	600.00
2-Hydroxynapthalene	227.07	450.46	947.49
3-Hydroxyfluorene	4.51	7.71	20.09
2-Hydroxyfluorene	12.64	20.39	47.80
1-Hydroxyphenanthrene	7.79	12.19	20.07
1-Hydroxypyrene	6.60	11.27	20.34

Table S2 The percentiles of the urinary PAHs in both creatinine adjusted and unadjusted in study about self-reported trouble sleeping.

Variable	25th percentile	50th percentile	75th percentile
**Unadjusted urinary PAHs (μg/L)**			
1-Hydroxynapthalene	736.00	1770.00	6360.30
2-Hydroxynapthalene	2042.20	4704.00	10700.00
3-Hydroxyfluorene	40.00	84.30	255.00
2-Hydroxyfluorene	111.00	231.20	578.00
1-Hydroxyphenanthrene	67.00	125.00	232.00
1-Hydroxypyrene	49.50	110.10	227.00
**Creatinine adjusted urinary PAHs (μg/mg creatinine)**			
1-Hydroxynapthalene	77.86	170.11	601.58
2-Hydroxynapthalene	227.35	450.56	948.42
3-Hydroxyfluorene	4.51	7.73	22.12
2-Hydroxyfluorene	12.66	20.40	48.00
1-Hydroxyphenanthrene	7.80	12.20	20.07
1-Hydroxypyrene	6.60	11.27	20.35

Table S3. Sensitivity analysis for exploring relationship between log-transformed urinary PAH metabolites and prevalence of short sleep duration in participants without chronic conditions.

Chemicals (μg/mg creatinine)	log-transformed urinary PAH metabolites	P-value	Q1 OR (95%CI)	Q4 OR (95%CI)	$P_{\text{for trend}}$
**1-Hydroxynapthalene**	1.09(1.04,1.15)	**0.001**	1.00 (reference)	1.49(1.22,1.81)	**0.002**
**2-Hydroxynapthalene**	1.20(1.10,1.31)	**<0.001**	1.00 (reference)	1.52(1.21,1.90)	**<0.001**
**3-Hydroxyfluorene**	1.16(1.08,1.24)	**<0.0001**	1.00 (reference)	1.46(1.15,1.84)	**0.013**
**2-Hydroxyfluorene**	1.22(1.12,1.32)	**<0.0001**	1.00 (reference)	1.62(1.29,2.02	**<0.001**
**1-Hydroxyphenanthrene**	1.19(1.05,1.34)	**0.01**	1.00 (reference)	1.48(1.16,1.89)	**0.002**
**1-Hydroxypyrene**	1.10(1.00,1.20)	**0.05**	1.00 (reference)	1.29(1.00,1.67)	**0.029**

Model were adjusted for age, gender, race/ethnicity, educational status, family poverty income ratio, and general health; OR: odds ratio; CI, confidence interval.

Table S4. Sensitivity analysis for exploring relationship between log-transformed urinary PAH metabolites and prevalence of self-reported trouble sleeping in participants without chronic conditions.

Chemicals (μg/mg creatinine)	log-transformed urinary PAH metabolites	P-value	Q1 OR (95%CI)	Q4 OR (95%CI)	$P_{\text{for trend}}$
**1-Hydroxynapthalene**	1.13(1.06,1.21)	**<0.001**	1.00 (reference)	1.54(1.15,2.05)	**0.006**
**2-Hydroxynapthalene**	1.16(1.04,1.30)	**0.01**	1.00 (reference)	1.39(1.02,1.89)	**0.03**
**3-Hydroxyfluorene**	1.13(1.04,1.22)	**0.003**	1.00 (reference)	1.44(1.09,1.92)	**0.01**
**2-Hydroxyfluorene**	1.14(1.04,1.25)	**0.01**	1.00 (reference)	1.27(0.99,1.63)	0.06
**1-Hydroxyphenanthrene**	1.13(1.00,1.27)	**0.05**	1.00 (reference)	1.28(0.96,1.73)	0.10
**1-Hydroxypyrene**	1.15(1.02,1.29)	**0.02**	1.00 (reference)	1.42(1.06,1.92)	**0.02**

Model were adjusted for age, gender, race/ethnicity, educational status, family poverty income ratio, and general health; OR: odds ratio; CI, confidence interval.

Table S5. Sensitivity analysis for exploring relationship between log-transformed urinary PAH metabolites and prevalence of short sleep duration in 2005-2010 and 2011-2016 NHANES waves.

Chemicals (μg/mg creatinine)	log-transformed urinary PAH metabolites	P-value	Q1 OR (95%CI)	Q4 OR (95%CI)	P for trend
**2005-2010 NHANES waves**					
1-Hydroxynapthalene	1.06(1.01,1.10)	**0.01**	1.00 (reference)	1.15(0.96,1.37)	0.058
2-Hydroxynapthalene	1.14(1.05,1.25)	**0.003**	1.00 (reference)	1.38(1.06,1.79)	**0.021**
3-Hydroxyfluorene	1.11(1.04,1.19)	**0.004**	1.00 (reference)	1.22(0.93,1.59)	0.203
2-Hydroxyfluorene	1.15(1.06,1.25)	**0.001**	1.00 (reference)	1.33(1.07,1.65)	**0.007**
1-Hydroxyphenanthrene	1.15(1.04,1.28)	**0.01**	1.00 (reference)	1.29(1.05,1.57)	**0.001**
1-Hydroxypyrene	1.15(1.05,1.27)	**0.004**	1.00 (reference)	1.51(1.20,1.89)	**<0.001**
**2011-2016 NHANES waves**					
1-Hydroxynapthalene	1.08(1.02,1.14)	**0.01**	1.00 (reference)	1.45(1.17,1.80)	**0.022**
2-Hydroxynapthalene	1.19(1.07,1.33)	**0.002**	1.00 (reference)	1.58(1.15,2.16)	**0.006**
3-Hydroxyfluorene	1.11(1.03,1.20)	**0.01**	1.00 (reference)	1.27(1.01,1.59)	0.081
2-Hydroxyfluorene	1.16(1.06,1.27)	**0.001**	1.00 (reference)	1.45(1.14,1.85)	**0.014**
1-Hydroxyphenanthrene	1.11(0.99,1.25)	0.08	1.00 (reference)	1.37(1.08,1.74)	**0.011**
1-Hydroxypyrene	1.07(0.97,1.17)	0.18	1.00 (reference)	1.17(0.89,1.52)	0.274

Model were adjusted for age, gender, race/ethnicity, educational status, family poverty income ratio, and general health; OR: odds ratio; CI, confidence interval.

Table S6. Sensitivity analysis for exploring relationship between log-transformed urinary PAH metabolites and prevalence of self-reported trouble sleeping in 2005-2010 and 2011-2016 NHANES waves.

Chemicals (μg/mg creatinine)	log-transformed urinary PAH metabolites	P-value	Q1 OR (95%CI)	Q4 OR (95%CI)	P for trend
**2005-2010 NHANES waves**					
1-Hydroxynapthalene	1.13(1.07,1.19)	**<0.0001**	1.00 (reference)	1.43(1.11,1.83)	**0.004**
2-Hydroxynapthalene	1.16(1.07,1.26)	**<0.001**	1.00 (reference)	1.45(1.13,1.86)	**0.005**
3-Hydroxyfluorene	1.12(1.04,1.21)	**0.004**	1.00 (reference)	1.28(0.98,1.66)	0.061
2-Hydroxyfluorene	1.15(1.05,1.26)	**0.004**	1.00 (reference)	1.28(0.98,1.69)	**0.037**
1-Hydroxyphenanthrene	1.15(1.03,1.29)	**0.01**	1.00 (reference)	1.33(1.05,1.69)	**0.016**
1-Hydroxypyrene	1.08(0.97,1.21)	0.15	1.00 (reference)	1.19(0.89,1.59)	0.493
**2011-2016 NHANES waves**					
1-Hydroxynapthalene	1.13(1.07,1.20)	**<0.0001**	1.00 (reference)	1.57(1.18,2.08)	**0.002**
2-Hydroxynapthalene	1.21(1.09,1.35)	**0.001**	1.00 (reference)	1.47(1.08,2.00)	**0.008**
3-Hydroxyfluorene	1.13(1.06,1.20)	**<0.001**	1.00 (reference)	1.42(1.10,1.83)	**0.008**
2-Hydroxyfluorene	1.16(1.08,1.25)	**<0.001**	1.00 (reference)	1.44(1.18,1.75)	**0.003**
1-Hydroxyphenanthrene	1.08(0.97,1.20)	0.16	1.00 (reference)	1.15(0.86,1.54)	0.272
1-Hydroxypyrene	1.15(1.02,1.28)	**0.02**	1.00 (reference)	1.52(1.15,2.00)	**0.016**

Model were adjusted for age, gender, race/ethnicity, educational status, family poverty income ratio, and general health; OR: odds ratio; CI, confidence interval.

Figure S1. Directed acyclic graph of the associations between PAH exposure and sleep problems.

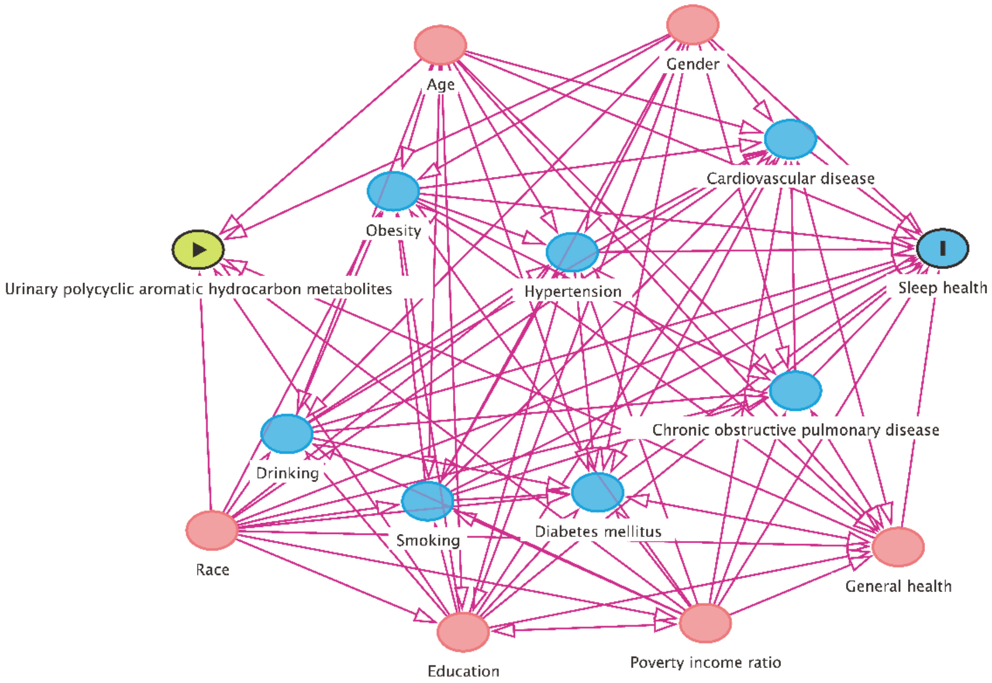

Figure S2. Cubic splines for the associations of urinary PAH metabolites with the prevalence of self-reported trouble sleeping. Model adjusted for age, gender, race/ethnicity, educational status, family poverty income ratio, and general health.

A. 1-Hydroxynapthalene;

B. 2-Hydroxynapthalene;

C. 3-Hydroxyfluorene;

D. 2- Hydroxyfluorene;

E. 1-Hydroxyphenanthrene;

F. 1-Hydroxypyrene.

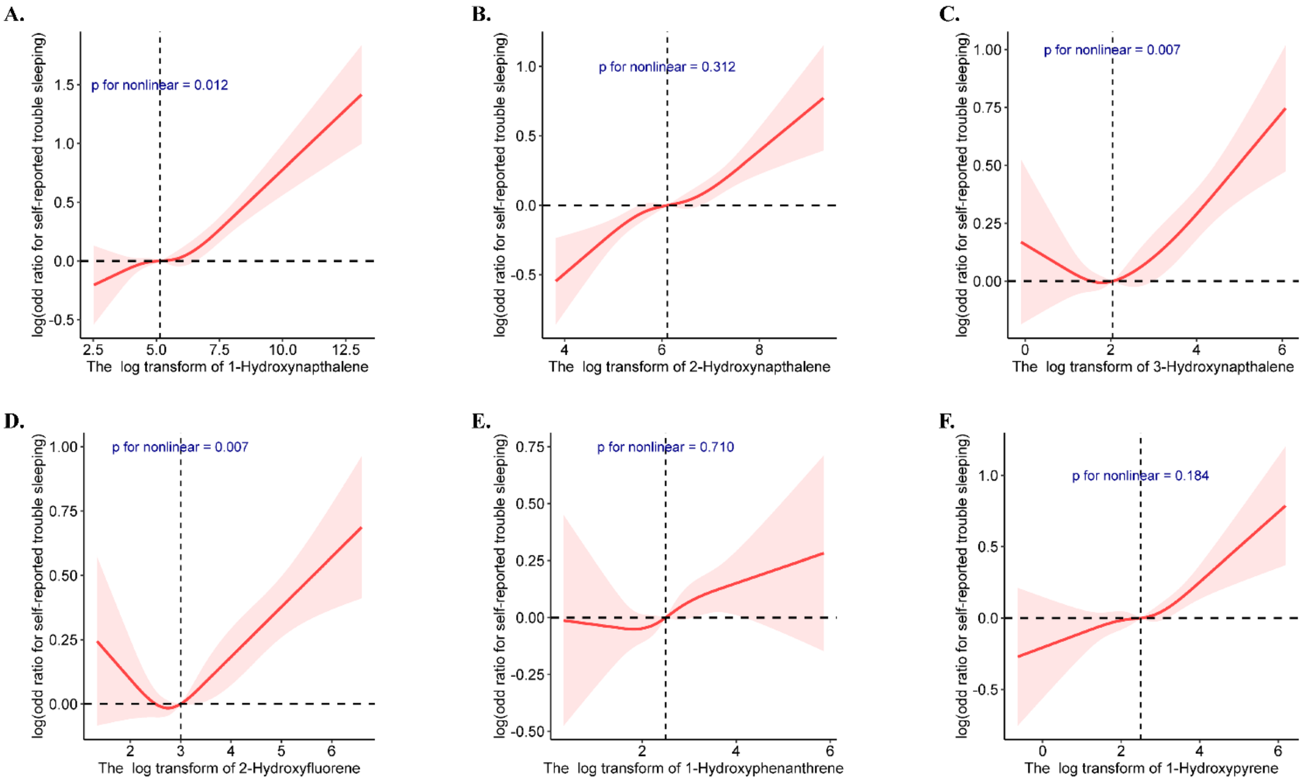

Supplement: Supplementary file 1 [file Data_Sheet_1.pdf]
